# Functional analysis of *COP1* and *SPA* orthologs from Physcomitrella and rice during photomorphogenesis of transgenic Arabidopsis reveals distinct evolutionary conservation

**DOI:** 10.1186/1471-2229-14-178

**Published:** 2014-07-01

**Authors:** Aashish Ranjan, Stephen Dickopf, Kristian K Ullrich, Stefan A Rensing, Ute Hoecker

**Affiliations:** 1 Botanical Institute and Cluster of Excellence on Plant Sciences (CEPLAS), Biocenter, University of Cologne, Zülpicher Str. 47b, 50674 Cologne, Germany; 2 Plant Cell Biology, Faculty of Biology, University of Marburg, Karl-von-Frisch-Str. 8, 35043 Marburg, Germany; 3 Present addresss: Life Sciences Addition #2237, Section of Plant Biology, UC Davis, One Shields Ave, Davis, CA 95616, USA

**Keywords:** Photomorphogenesis, Light signal transduction, Flowering time, COP1, SPA1, Evolution, Physcomitrella, Rice, Arabidopsis

## Abstract

**Background:**

Plants have evolved light sensing mechanisms to optimally adapt their growth and development to the ambient light environment. The COP1/SPA complex is a key negative regulator of light signaling in the well-studied dicot *Arabidopsis thaliana*. COP1 and members of the four SPA proteins are part of an E3 ubiquitin ligase that acts in darkness to ubiquitinate several transcription factors involved in light responses, thereby targeting them for degradation by the proteasome. While COP1 is also found in humans, SPA proteins appear specific to plants. Here, we have functionally addressed evolutionary conservation of *COP1* and *SPA* orthologs from the moss Physcomitrella, the monocot rice and the dicot Arabidopsis.

**Results:**

To this end, we analyzed the activities of COP1- and SPA-like proteins from *Physcomitrella patens* and rice when expressed in Arabidopsis. Expression of rice COP1 and Physcomitrella COP1 protein sequences predominantly complemented all phenotypic aspects of the viable, hypomorphic *cop1-4* mutant and the null, seedling-lethal *cop1-5* mutant of Arabidopsis: rice COP1 fully rescued the constitutive-photomorphogenesis phenotype in darkness and the leaf expansion defect of *cop1* mutants, while it partially restored normal photoperiodic flowering in *cop1*. Physcomitrella COP1 partially restored normal seedling growth and flowering time, while it fully restored normal leaf expansion in the *cop1* mutants. In contrast, expression of a SPA ortholog from Physcomitrella (PpSPAb) in Arabidopsis *spa* mutants did not rescue any facet of the *spa* mutant phenotype, suggesting that the PpSPAb protein is not functionally conserved or that the Arabidopsis function evolved after the split of mosses and seed plants. The SPA1 ortholog from rice (OsSPA1) rescued the *spa* mutant phenotype in dark-grown seedlings, but did not complement any *spa* mutant phenotype in light-grown seedlings or in adult plants.

**Conclusion:**

Our results show that COP1 protein sequences from Physcomitrella, rice and Arabidopsis have been functionally conserved during evolution, while the SPA proteins showed considerable functional divergence. This may - at least in part - reflect the fact that *COP1* is a single copy gene in seed plants, while SPA proteins are encoded by a small gene family of two to four members with possibly sub- or neofunctionalized tasks.

## Background

Since plants use sunlight as their primary source of energy they have evolved mechanisms of light sensing in order to optimally adjust their growth and development accordingly. Light-adapted responses are particularly obvious during seedling growth. Dark-grown seedlings usually exist under soil cover and therefore respond with etiolation, showing a long hypocotyl, small and closed cotyledons, an apical hook and a lack of chlorophyll synthesis. Light-grown seedlings, in contrast, are green and exhibit a short hypocotyl, open, expanded and green cotyledons and no apical hook. Other light-induced responses include phototropism, leaf expansion, the shade avoidance response and photoperiodic flowering [1,2]. To sense the light, plants have several classes of photoreceptors: the red (R) and far-red (FR) sensing phytochromes, the blue (B)/UV-A responsive cryptochromes, phototropins and ZEITLUPE family members and the recently identified UV-B sensing UV-RESISTANCE LOCUS 8 (UVR8) protein [3-6].

The molecular events during light signal transduction are best understood in the model species Arabidopsis. After activation by light, phytochrome and cryptochrome photoreceptors inhibit the activity of a key negative regulator of light signal transduction, the CULLIN4 (CUL4)-dependent E3 ubiquitin ligase complex CONSTITUTIVELY PHOTOMORPHOGENIC1/SUPPRESSOR OF PHYA-105 (COP1/SPA). In darkness, COP1/SPA acts to ubiquitinate activators of the light response, such as the transcription factors ELONGATED HYPOCOTYL5 (HY5), LONG HYPOCOTYL IN FR 1 (HFR1), B-BOX DOMAIN PROTEINS (BBX) proteins, PRODUCTION OF ANTHOCYANIN PIGMENT1 (PAP1) and PAP2 as well as several photoreceptors, thereby targeting them for degradation in the proteasome. In light-grown plants, in contrast, COP1/SPA activity is suppressed and the target proteins can accumulate and mediate light-regulated gene expression and photomorphogenesis [7-11]. Hence, mutants defective in *COP1* or in all four members of the *SPA* gene family show constitutive photomorphogenesis, exhibiting features of light-grown seedlings in complete darkness [12,13]. Besides controling seedling growth in response to light, the COP1/SPA complex is involved in multiple other light-induced responses, such as anthocyanin biosynthesis, leaf expansion, shade avoidance responses and photoperiodic flowering [7,11,14-19]. COP1/SPA also acts downstream of the UV-B receptor UVR8, but in contrast to R and B signaling - where COP1 acts as a repressor of light signaling - COP1/SPA functions as a positive regulator of the UV-B response [20].

The COP1/SPA complex likely forms a tetramer with two COP1 and two SPA proteins. COP1 and SPA proteins interact with each other via their respective coiled-coil domains [21-24]. COP1 and the four SPA proteins (SPA1-SPA4) share further structural similarity in that they contain related C-terminal WD-repeat domains which have dual roles in substrate recruitment and binding of DAMAGED DNA-BINDING PROTEIN1 (DDB1) of the CUL4 complex [11]. In their N-termini, COP1 and SPA proteins have distinct sequences, with COP1 containing a RING finger domain and SPA proteins carrying a kinase-like domain [25,26]. The mechanisms involved in light-mediated inhibition of COP1/SPA activity are not well understood but likely involve light-induced interaction of cryptochromes with SPA1, light-induced degradation of SPA1 and SPA2 as well as light-mediated nuclear exclusion of COP1 [27-33].

The four SPA proteins share highest sequence similarity to each other in their WD-repeat domain. Sequence conservation of the N-terminal domain is relatively low and mostly limited to the kinase-like domain. Based on sequence similarity, the four SPA proteins fall into two subgroups with SPA1 and SPA2 forming one subgroup and SPA3 and SPA4 forming the other subgroup [13]. Genetic analysis of *spa* mutants indicated that the four *SPA* genes have partly redundant but also distinct functions in plant growth and development [13,27,34].

COP1 functions have also been described in other flowering plant species. In rice, the *COP1* ortholog *PETER PAN SYNDROME1* (*PPS*) shortens the juvenile phase, a phenotype not reported for Arabidopsis, and delays flowering in short and long day [35]. The *COP1* ortholog of pea, *LIGHT-INDEPENDENT PHOTOMORHOGENESIS1* (*LIP1*), regulates seedling growth by affecting gibberellic acid levels [36,37]. In apple, *MdCOP1* affects anthocyanin levels in the fruit peel [9]. *COP1* also exists in non-plant lineages, e.g. humans, where hCOP1 acts as an E3 ubiquitin ligase to control the protein stability of a number of transcription factors, e.g. p53 or cJun [38]. *SPA* genes, in contrast, appear to be specific to plants, which indicates that human COP1 functions without a need for SPA proteins. This suggests that *SPA* genes might have evolved to place COP1 activity under the control of light. Indeed, the N-terminus of SPA1 was shown to be involved in the blue-light dependent interaction of SPA1 with cryptochrome photoreceptors [31,32].

Whole genome sequencing has shown that *COP1* and *SPA* genes exist in early diverged land plants, such as in the moss *Physcomitrella patens*. There are a number of light responses known in Physcomitrella, such as chloroplast movement, phototropism, caulonema branching and gametophore growth [39] as well as UV-B responses akin to those in Arabidopsis [40]. While *COP1* is a single copy gene in rice and Arabidopsis [11], genome sequence information predicted a total of nine paralogs in *P. patens*[41,42]. Both the rice and Physcomitrella genomes contain two *SPA*-related genes each [41-43]. Physcomitrella has functional phytochrome and cryptochrome photoreceptors [39,44-47], allowing the possibility that *PpCOP1* and *PpSPA* genes may also function in light signal transduction in Physcomitrella.

To address the evolutionary conservation of COP1 and SPA protein sequences, we expressed *COP1* and *SPA* coding sequences from rice and Physcomitrella in the respective *cop1* and *spa* mutant backgrounds of Arabidopsis. Our results show that COP1 sequences are functionally much more conserved than SPA sequences, suggesting that gene duplication of *SPA* genes in the flowering plant lineage has contributed to divergence of *SPA* gene functions.

## Results

### A comparison of Physcomitrella, rice and Arabidopsis COP1 and SPA protein sequences

**Figure 1:**
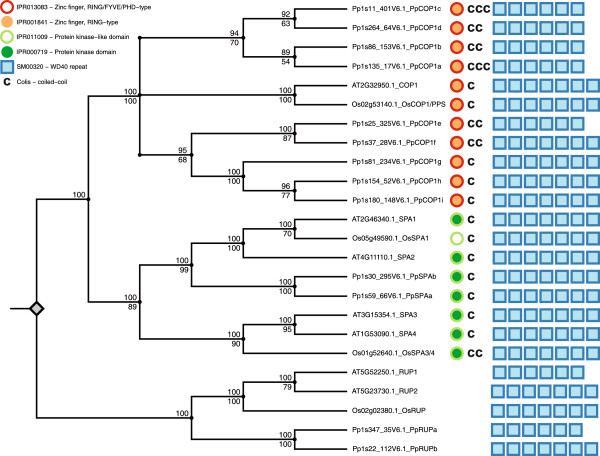
**Cladogram representing the COP1 and SPA gene family phylogeny in Arabidopsis, rice and Physcomitrella and overview of their protein domain structure.** The cladogram combines the phylogenetic relationships between the species analyzed which were obtained by Bayesian inference and maximum likelihood. Branch lengths are not in proportion to evolutionary times. Grey diamond represents root of the phylogeny set by the RUP gene family as an outgroup. Numbers on internal branches indicate Bayesian inference prosterior probabilities (support values) in percent (upper number) or maximum likeliood bootstrap support values in percent (lower number). Next to each protein name obtained by the used sequence databases an alias was attached. Protein domains important for COP1 and SPA gene function obtained by InterProScan5 were plotted next to each protein; red rings, IPR013083 - Zinc finger, RING/FYVE/PHD - type; orange circles, IPR001841 - Zinc finger, RING - type; light green rings, IPR011009 - Protein kinase - like domain; green circles, IPR000719 - Protein kinase domain; blue boxes represent number of WD40 repeats, SM00320 - WD40 repeat; “c” symbols represent number of coiled-coil occurrence based on Coils prediction. Detailed settings used for tree construction and tree plotting can be obtained from the methods chapter.

Based on the v1.6 genome annotation currently available [48], the Physcomitrella genome contains 9 *COP1*-like genes (Figure 1; Additional file 1: Figure S1), as was predicted previously based on v1.2 [41]. The predicted PpCOP1 protein sequences share 61-82% amino acid sequence identity among each other and 55-64% amino acid sequence identity with the Arabidopsis COP1 protein. The COP1 ortholog from rice (PPS [35], here for clarity from now on referred to as OsCOP1) and Arabidopsis COP1 share approx. 70% identical amino acids. Like Arabidopsis COP1, all predicted PpCOP1 proteins and OsCOP1 contain a RING finger motif, at least one coiled-coil domain and a WD40 repeat domain (Figure 1; Additional file 1: Figure S1C; Additional file 2: Figure S2, Additional file 3: Figure S3).

While the *COP1* gene family has expanded in Physcomitrella as compared to a single *COP1* gene reported in flowering plant species, there are only two predicted *SPA* genes in Physcomitrella. These two *PpSPA* genes are very similar to each other (89% amino acid identity of the predicted proteins), suggesting that they represent recent duplication events based on an ortholog of AtSPA1/2 (Figure 1; Additional file 1: Figure S1A, B; Additional file 4: Figure S4). We named the two Physcomitrella *SPA* genes *PpSPAa* (Pp1s59_66V6.1) and *PpSPAb* (Pp1s30_295V6.1). There are two predicted rice SPA proteins of which each groups with one subclass from Arabidopsis (AtSPA1/2, AtSPA3/4) (Figure 1; Additional file 1: Figure S1A, B), evidencing that two paralogs were already present in the last common ancestor of monocots and dicots. The SPA1/SPA2-like rice SPA was more similar to Arabidopsis SPA1 than to Arabidopsis SPA2. We therefore refer to this rice SPA as rice SPA1-like or OsSPA1 (Os05g49590.1). The predicted SPA3/SPA4-like SPA from rice equally resembles Arabidopsis SPA3 and SPA4 protein sequences. We therefore refer to it as rice SPA3/4-like or OsSPA3/4 (Os01g52640.1). The predicted domain structures of Physcomitrella and rice SPA proteins are similar to those from Arabidopsis SPA proteins: they all contain an N-terminal kinase-like domain, a coiled-coil domain and seven WD40-repeats (Figure 1; Additional file 1: Figure S1C, Additional file 3: Figure S3, Additional file 4: Figure S4). Similar to Arabidopsis SPA proteins, the kinase-like domains from rice and Physcomitrella SPA proteins share only limited sequence conservation with *bona fide* Ser/Thr kinase consensus motifs because amino acid residues that are normally highly conserved in Ser/Thr kinases are not conserved in PpSPA and OsSPA proteins. Nevertheless, sequences in the kinase-like domain that are conserved among the four Arabidopsis SPA proteins are also highly conserved in OsSPA and PpSPA proteins (Additional file 4: Figure S4). All SPA sequences in Arabidopsis, rice and Physcomitrella contain a predicted coiled-coil domain (Additional file 3: Figure S3), though the sequence of the respective coiled-coil domain is not strongly conserved among Arabidopsis, rice or Physcomitrella SPA proteins. This suggests a structural rather than sequence-based conservation of this domain in the SPA proteins. The SPA protein sequences are most conserved within the WD40-repeat domain, with Physcomitrella SPAa and SPAb showing 65% amino acid identity with AtSPA1 - compared with 42% when aligning the complete protein sequences.

Rice and Physcomitrella also contain predicted orthologs of the Arabidopsis *RUP* genes. Arabidopsis RUP proteins consist of COP1/SPA-like WD40 repeats and function as negative regulators of UV-B signaling [49,50]. The rice genome contains 1 ortholog of RUP, while Physcomitrella has two predicted RUPs (Figure 1; Additional file 1: Figure S1, Additional file 5: Figure S5).

### Functional analysis of COP1-like proteins from rice and Physcomitrella in the hypomorphic *cop1-4* mutant of Arabidopsis

In order to address the evolutionary conservation of COP1 and SPA function, we expressed the coding sequence of Physcomitrella, rice and - as a control - Arabidopsis *COP1* and *SPA* genes in transgenic Arabidopsis *cop1* and *spa* mutants, respectively, to subsequently evaluate whether the transgenes complement the respective mutant phenotypes. Though protein detection in the transgenic plants is desirable, we did not add an epitope tag to the coding sequence because a tag might negatively affect protein function. Among the nine *PpCOP1* genes, we chose the one with the highest sequence similarity to *AtCOP1*, based on BLAST scores, for the complementation study (Pp1s135_17V6.1, PpCOP1a, Figure 1). The coding sequences of *OsCOP1, PpCOP1a* and *AtCOP1* were placed under the control of the *35S* constitutive promoter and introduced into the hypomorphic *cop1-4* mutant and into the *cop1-5* null mutant of Arabidopsis. While the *cop1* null mutant is seedling lethal, the *cop1-4* mutant is viable, producing a truncated COP1 protein lacking the C-terminal WD-repeat domain [12,51].

**Figure 2:**
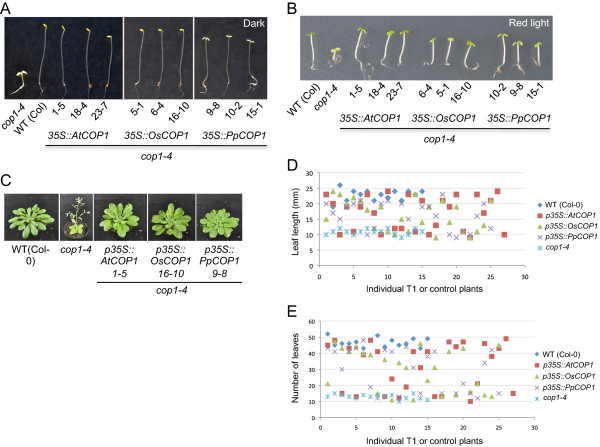
**Complementation analysis of Arabidopsis *cop1-4 *hypomorphic mutants carrying the rice, Physcomitrella or Arabidopsis *COP1 *transgene. A,B.** Visual phenotype of *cop1-4* mutant Arabidopsis seedlings that are homozygous for the transgenes *AtCOP1* (Arabidopsis *COP1*), *OsCOP1* (rice *COP1*) or *PpCOP1a* (Physcomitrella *COP1*). Seedlings were grown in darkness **(A)** or red light (**B,** 5 μmol m^-2^ s^-1^) for four days. Three independent transgenic lines and, as controls, wildtype Col (WT) and a *cop1-4* mutant are shown. **C.** Visual phenotype of *cop1-4* mutant Arabidopsis plants. Genotypes were as in **B.** Plants were grown in short day for four weeks. **D,E.** Scatter plot representing leaf length **(D)** and flowering time **(E)** of 25–27 individual, i.e. independent T1 primary transformants and 15 individual wild-type and *cop1-4* mutant control plants. Plants were grown in short day.

*cop1-4* mutant seedlings undergo constitutive photomorphogenesis in darkness, exhibiting short hypocotyls and open cotyledons (Figure 2A [51]). Transgenic *cop1-4* seedlings expressing the Arabidopsis *COP1* gene or rice *COP1* ortholog fully etiolated in darkness and thus resembled the wild type. Hence, *AtCOP1* and *OsCOP1* fully complemented the *cop1-4* mutant phenotype in darkness. Transgenic *cop1-4* seedlings carrying the *PpCOP1a* transgene showed a partial rescue of the *cop1-4* mutant phenotype in darkness: *PpCOP1a* lines exhibited a longer hypocotyl than *cop1-4* in darkness but failed to fully etiolate, as indicated by the open cotyledons and the lack of an apical hook (Figure 2A). Of 25 independent *PpCOP1a* lines investigated, none showed a full rescue of the *cop1-4* mutant phenotype in darkness. When grown in light of low to intermediate fluence rates, *cop1-4* mutant seedlings exhibited a shorter hypocotyl than the wild type ([51], Figure 2B). This mutant phenotype was similarly complemented by all three transgenes, *AtCOP1*, *OsCOP1* and *PpCOP1a* (Figure 2B).

Besides the constitutive photomorphogenesis in seedlings, *cop1-4* mutants exhibit mutant phenotypes in the adult plant: *cop1-4* mutant plants are small and dwarfed and they flower earlier than the wild type, particularly under short day conditions [51]. Transgenic *AtCOP1*, *OsCOP1* and *PpCOP1a cop1-4* mutant lines were similar in size as the wild type and flowered at a similar time as the wild type (Figure 2C,D,E). For each of the three transgenes, about half of the transgenic T1 plants showed full rescue of the *cop1-4* mutant adult phenotypes (Figure 2D,E). Hence, *OsCOP1* and *PpCOP1a*, like *AtCOP1*, were able to fully complement the *cop1-4* mutant phenotypes in adult plants.

### Functional analysis of COP1–like proteins from rice and Physcomitrella in the *cop1-5* null mutant of Arabidopsis

**Figure 3:**
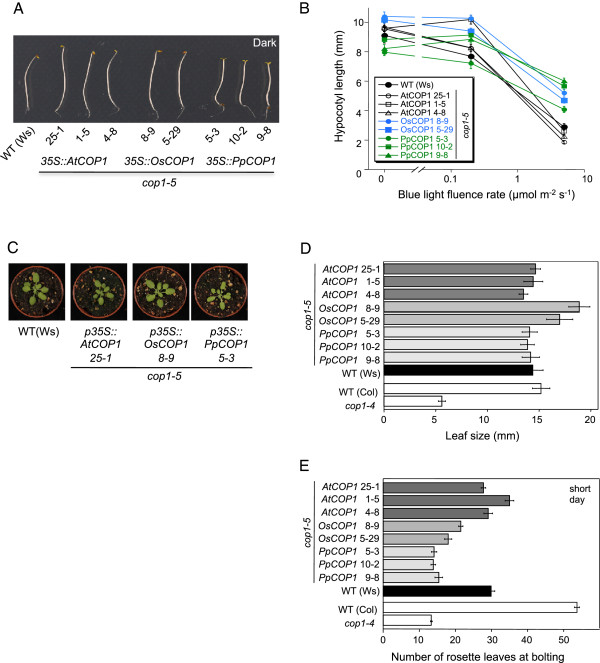
**Complementation analysis of Arabidopsis *cop1-5 *null mutants carrying the rice, Physcomitrella or Arabidopsis *COP1 *transgene. A.** Visual phenotype of *cop1-5* null mutant Arabidopsis seedlings that are homozygous for the transgenes *AtCOP1*, *OsCOP1* or *PpCOP1a*. Seedlings were grown in darkness for four days. WT (Ws) and three independent transgenic lines are shown. *cop1-5* mutant seeds failed to germinate due to the seedling-lethal phenotype and are therefore not shown. **B.** Hypocotyl elongation response of transgenic *cop1-5* mutant seedlings to blue light. Genotypes were as in **A.** Error bars show the standard error of the mean (SEM). **C.** Visual phenotype of transgenic *cop1-5* mutant Arabidopsis plants. Genotypes were as in A; one representative transgenic line is shown for each transgene. Plants were grown in short day for three weeks. **D,E.** Leaf size **(D)** and flowering time **(E)** of homozygous transgenic *cop1-5* lines. Genotypes were as in **A.** Two to three independent transgenic lines are shown for each construct. Wild type (Ws) serves as a control. The *cop1-5* mutant is seedling-lethal and therefore not shown. Rather, *cop1-4* and WT (Col) are shown as controls to allow evaluation of growth conditions. Error bars show the SEM, n = 12.

Since the *cop1-4* mutant allele expresses a truncated COP1 protein retaining the N-terminal part of COP1 including the coiled-coil domain [51], rescue of the *cop1-4* mutant phenotype by expression of *OsCOP1* or *PpCOP1a* might depend on the presence of the truncated COP1-4 protein, especially since the retained coiled-coil domain might allow protein-protein interaction with OsCOP1 and PpCOP1a. We therefore introduced the transgenes also into the *cop1-5* null mutant background by transforming *cop1-5/+*plants and by crossing transgenic *cop1-4* mutants with *cop1-5/+*plants. Homozygous *cop1-5* (-/-) mutant seeds in the progeny could be easily recognized by their black seed color, though they mostly failed to germinate [51]. Assuming Mendelian segregation of the seedling-lethal *cop1-5* mutant phenotype, the penotypic effect of the transgenes should be analyzable in the respective T2 generations based on the segregation ratio of mutant and wild-type phenotypes. However, we found a much reduced transmission frequency of the *cop1-5* mutant allele when compared to the *COP1* wild-type allele, thus making the analysis of segregating populations ambiguous. We therefore generated homozygous *cop1-5* mutant lines that were also homozygous for the respective transgene. Figure 3A shows that *AtCOP1* and *OsCOP1* fully restored a wild-type phenotype in dark-grown homozygous *cop1-5* mutant seedlings. Hence, the *AtCOP1* and *OsCOP1* transgenes not only rescued the seedling-lethal phenotype of *cop1-5* but also fully complemented its *fusca* phenotype of constitutive photomorphogenesis and strong anthocyanin production which was described for strong *cop1* alleles [51]. *PpCOP1a cop1-5* seedlings, in contrast, showed open cotyledons and a slightly shorter hypocotyl than the wild type when grown in darkness (Figure 3A,B). Thus, expression of *PpCOP1a* resulted in partial complementation of the *cop1-5* mutant phenotype. In light-grown seedlings, the control construct *AtCOP1* fully complemented the *cop1-5* mutant phenotype. In contrast, B- and FR-grown *OsCOP1 cop1-5* and *PpCOP1a cop1-5* seedlings were even taller than wild-type seedlings, especially at higher fluence rates, indicating a reduced response to B and FR when compared to the wild type (Figure 3B; Additional file 6: Figure S6). In R, all transgenic seedlings behaved similar to the wild type (Additional file 6: Figure S6).

Since all three transgenes rescued the seedling-lethal phenotype of *cop1-5*, we were able to analyze the activity of the transgene also in the adult stage. Transgenic *OsCOP1 cop1-5*, *PpCOP1a cop1-5* and *AtCOP1 cop1-5* plants were of similar size as the wild type (Figure 2C,D). With respect to flowering time, transgenic *AtCOP1 cop1-5* lines flowered at a similar time as the Ws wild type while transgenic *OsCOP1 cop1-5* and, in particular, *PpCOP1a cop1-5* lines flowered earlier than the wild type and the *AtCOP1 cop1-5* transgenic lines (Figure 2E). These results indicate that the COP1 sequences from rice and Physcomitrella only partially rescued this aspect of the *cop1-5* mutant phenotype.

### Rice and Physcomitrella SPA protein-coding sequences do not complement the light hypersensitivity-phenotype of the Arabidopsis *spa1 spa3 spa4* triple mutant

To analyze functional conservation of rice and Physcomitrella SPA1-related protein-coding sequences we expressed *OsSPA1* and *PpSPAb* ORFs in an Arabidopsis *spa* mutant. The two Physcomitrella SPA proteins, SPAa and SPAb are highly similar to each other (89% amino acid sequence identity) and both share equal sequence similarity to the Arabidopsis SPA1. We therefore chose only one of these SPAs, SPAb*,* for our analyses. As controls, we included the Arabidopsis *SPA1* and *SPA4* ORFs because these two SPAs are representative for the partially distinct functions of the four *SPA* genes [13,15,34]. We transformed these constructs into the *spa1 spa3 spa4* triple mutant because this mutant is a viable *spa* mutant showing defects in multiple phenotypes including seedling deetiolation, leaf expansion and flowering time control [13,15]. Initially, we expressed the *SPA* coding sequences under the control of the *35S* promoter. However, the Arabidopsis *35S::AtSPA1* and *35S::AtSPA4* constructs produced very low complementation rates (<10% of transgenic plants) in the *spa* triple mutant, an observation we had made before [52]. We therefore proceeded to express the respective *SPA* coding sequences under the control of the endogenous Arabidopsis *AtSPA1* and *AtSPA4* 5´ and 3´ regulatory sequences which previously produced very high complementation rates among transgenic *spa* mutant plants (>90%) [27,52]. For linguistic simplicity, we will refer to these regulatory sequences as ´promoters´ from now on.

**Figure 4:**
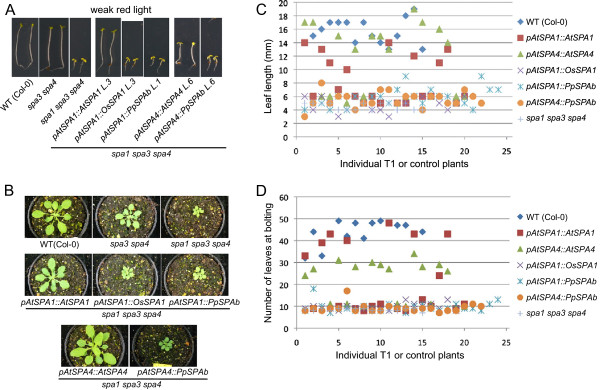
***SPA1 *orthologs from rice and Physcomitrella do not complement seedling nor adult phenotypes of Arabidopsis *spa1 spa3 spa4 *mutants in the light. A.** Visual phenotype of *spa1 spa3 spa4* mutant Arabidopsis seedlings that carry constructs with the coding sequence of Arabidopsis *AtSPA1* or *AtSPA4*, rice *OsSPA1* or Physcomitrella *PpSPAb* driven by the Arabidopsis *AtSPA1* or *AtSPA4* promoters (*pAtSPA1*, *pAtSPA4*). Seedlings were grown in weak red light (0.1 μmol m^-2^ s^-1^) for four days. **B.** Visual phenotype of plants grown in short day for four weeks. Genotypes are as in **A.C,D.** Scatter plot showing leaf length **(C)** and flowering time **(D)** of 18–24 individual, i.e. independent T1 primary transformants carrying the transgenes described in **(A)** and 15 individual wild-type and *spa1 spa3 spa4* mutant control plants. Plants were grown in short day.

*spa1 spa3 spa4* triple mutant seedlings etiolate normally in darkness, but have a severely reduced hypocotyl length in weak light when compared to the wild type. Hence, this mutant is strongly hypersensitive to light ([13], Figure 4A). Expression of *AtSPA1* from the *AtSPA1* promoter fully restored the *spa3 spa4* phenotype in the *spa1 spa3 spa4* mutant, thus reflecting the activity of the native *SPA1* gene. In contrast, expression of rice *OsSPA1* or Physcomitrella *PpSPAb* from the *AtSPA1* promoter did not alter the *spa1 spa3 spa4* mutant seedling phenotype in any of the 20 independent transgenic lines analyzed for each construct (Figure 4A). Similarly, when *PpSPAb* was expressed from the Arabidopsis *AtSPA4* promoter, no change in the *spa1 spa3 spa4* mutant phenotype was observed, while expression of the control construct *AtSPA4::AtSPA4* caused an elongation of the hypocotyl when compared to the *spa1 spa3 spa4* progenitor, though the effect of *AtSPA4::AtSPA4* was consistently weaker than that of *AtSPA1::AtSPA1*, as expected [13].

In the adult stage, none of the constructs containing the *OsSPA1* or *PpSPAb* coding sequences complemented the dwarfism or the early flowering time of the *spa1 spa3 spa4* mutant (Figure 4B,C,D). Expression of the control constructs *AtSPA1::AtSPA1* or *AtSPA4::AtSPA4*, in contrast, rescued these facets of the *spa1 spa3 spa4* mutant phenotype to the expected degree [13,15].

**Figure 5:**
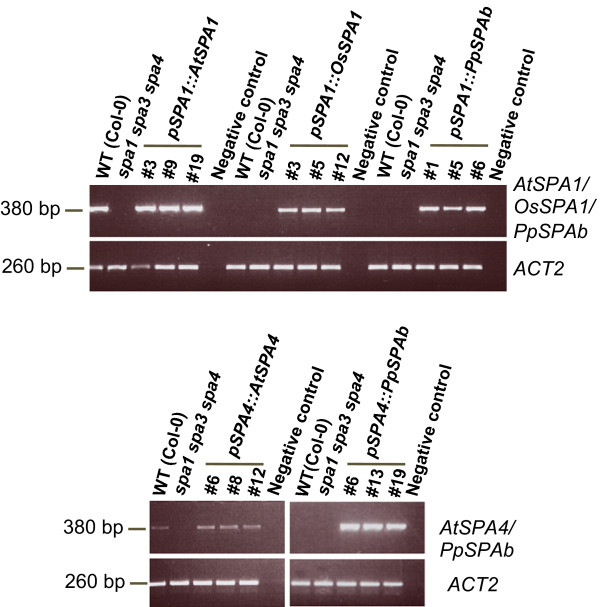
**Transcript levels of the transgenes in transgenic *spa1 spa3 spa4 *mutant lines. ***AtSPA1*, *OsSPA1, PpSPAb* and *AtSPA4* transcript levels in transgenic seedlings carrying the indicated constructs. Transcript levels were analyzed by semi-quantitative RT-PCR using primers specific for the respective transgene-encoded transcript. Seedlings used for RNA isolation were grown in weak red light (0.1 μmol m^-2^ s^-1^) for four days. Primers amplifying the *ACT2* transcript were used as a control.

To confirm that *OsSPA1* and *PpSPAb* genes are indeed expressed in the transgenic plants, we analyzed *SPA* transcript levels by semiquantitative RT-PCR. Figure 5 shows that all transgenes were expressed. This indicates that the failure of *OsSPA1* and *PpSPAb* coding sequences to complement the *spa* triple mutant phenotype was not caused by a lack of expression of the respective *SPA* genes.

### Functional analysis of *SPA* orthologs from rice and Physcomitrella in the constitutively photomorphogenic *spa1 spa2 spa3* mutant of Arabidopsis

**Figure 6:**
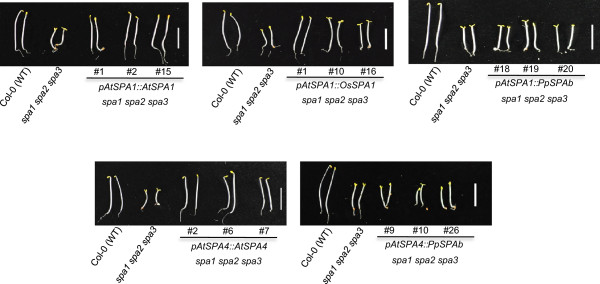
**Complementation analysis of dark-grown *spa1 spa2 spa3 *mutant seedlings carrying rice, Physcomitrella or Arabidopsis *SPA1 *or *SPA1*-related transgenes.** Visual phenotype of *spa1 spa2 spa3* mutant Arabidopsis seedlings that carry constructs with the coding sequence of Arabidopsis *AtSPA1*, rice *OsSPA1*, Physcomitrella *PpSPAb* or Arabidopsis *AtSPA4* driven by the Arabidopsis *SPA1* or *SPA4* promoters (*pAtSPA1*, *pAtSPA4*). Seedlings were grown in darkness for four days.

Since Arabidopsis *spa1 spa3 spa4* mutant seedlings analyzed above etiolate normally in darkness, this background precludes a genetic complementation analysis in dark-grown seedlings. We therefore introduced the *SPA* constructs also into the *spa1 spa2 spa3* triple mutant which undergoes constitutive seedling photomorphogenesis in darkness (Figure 6), while it develops normally as an adult plant [13,15].

**Figure 7:**
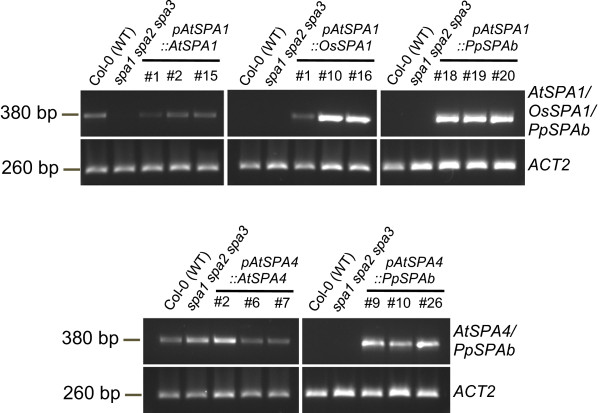
**Transcript levels of the transgenes in transgenic *spa1 spa2 spa3 *mutant lines. ***AtSPA1*, *OsSPA1, PpSPAb* and *AtSPA4* transcript levels in transgenic seedlings carrying the indicated constructs. Transcript levels were analyzed by semi-quantitative RT-PCR using primers specific for the respective transgene-encoded transcript. Seedlings used for RNA isolation were grown in darkness for four days. Primers amplifying the *ACT2* transcript were used as a control. Negative controls contained no template DNA.

Expression of the control constructs (*AtSPA1::AtSPA1; AtSPA4::AtSPA4*) fully complemented the *spa1 spa2 spa3* mutant phenotype in darkness: all of the *AtSPA1::AtSPA1* lines (12/12 independent lines total) and most of the *AtSPA4::AtSPA4* lines (10/11 total) exhibited normal skotomorphogenesis in darkness (Figure 6). When expressing the rice SPA1 (*AtSPA1::OsSPA1*), several transgenic lines showed partial (8/22 total) or full (1/22 total) complementation of the *spa1 spa2 spa3* mutant phenotype in darkness (Figure 6). Hence, OsSPA1 appears to be functional in Arabidopsis, though at a much reduced efficiency when compared to AtSPA1. In contrast, none of the 25 transgenic lines expressing Physcomitrella *PpSPAb* under the *AtSPA1* or *AtSPA4* promoters showed any rescue of the *spa1 spa2 spa3* mutant phenotype: these transgenic *spa1 spa3 spa4* seedlings underwent constitutive photomorphogenesis in darkness very similar to the *spa1 spa2 spa3* mutant progenitor (Figure 6). Hence, *PpSPAb* was non-functional in Arabidopsis. Again, all transgenes were expressed in the respective transgenic lines, as indicated by the presence of the transgene-encoded transcripts (Figure 7).

## Discussion

The COP1/SPA complex of Arabidopsis is a well-characterized key negative regulator that actively suppresses the light signaling cascade in dark-grown plants by ubiquitinating transcription factors which mediate the various light responses. The E3 ubiquitin ligase activity is conserved in the mammalian ortholog of COP1 which, however, appears to function without a need for SPA proteins since *SPA* genes appear to be specific to plants. SPA protein sequences are distinct from COP1 in that they carry a kinase-like domain in the N-terminus [13,26]. This kinase-like domain is conserved in Physcomitrella, rice and Arabidopsis SPA proteins and shows a similar divergence in sequence from *bona fide* Ser/Thr kinase motifs in all three species. This finding suggests on one hand that this kinase-like domain is of functional importance - though its exact role has so far remained elusive [31,32,34,53] - and on the other hand that early in land plant evolution this domain was already divergent in sequence from normal protein kinases.

Our functional analysis clearly shows that PpCOP1a from Physcomitrella is able to mostly replace the functions of COP1 in Arabidopsis. Similarly, rice OsCOP1 was able to mostly complement all aspects of the Arabidopsis *cop1* mutant phenotype. These findings suggest that COP1 is under strong negative selection in seed plants. Physcomitrella PpSPAb, in contrast, was incapable of complementing any of the *spa* mutant phenotypes in transgenic Arabidopsis, strongly suggesting that the PpSPAb protein is non-functional in Arabidopsis. Similarly, expression of the rice OsSPA1 protein in Arabidopsis *spa* mutants failed to complement any phenotypes of light-grown *spa* mutant plants and complemented the phenotype of dark-grown seedlings at a much reduced efficiency. These results suggest that SPA-like sequences underwent considerable functional divergence during evolution. However, since we cannot determine the PpSPAb and OsSPA1 protein levels in the transgenic Arabidopsis plants we cannot exclude the possibility that the apparent inactivity of PpSPAb and OsSPA1 in Arabidopsis are due to inefficient translation of the respective mRNAs or due to instability of the respective proteins in Arabidopsis when compared to the native Arabidopsis SPA1 protein. To fully understand the functional conservation between SPA1 from moss, rice and Arabidopsis, it will also be necessary to genetically identify OsSPA1 and PpSPA1 function in rice and Physcomitrella, respectively. Moreover, a protein-protein interaction analysis among the respective COP1 and SPA orthologs will be helpful in analyzing OsSPA1 and PpSPAb activity in Arabidopsis.

We can only speculate why the *COP1* gene appears to be subject to much less functional divergence than *SPA1*. One likely reason is the fact that *COP1* is a single-copy gene in flowering plants while SPA proteins are encoded by a small gene family comprising two to four members. Gene duplication is a powerful driving force of neo- and subfunctionalization during plant evolution [54]. The four *SPA* genes of Arabidopsis are indeed not fully redundant but have partially distinct functions during Arabidopsis development [13,15]. At least some of the functional divergence, the one between Arabidopsis *SPA1* and *SPA2*, has been mapped to the respective SPA protein sequence rather than the promoter sequences [27]. Hence, evidence strongly suggests that the four Arabidopsis SPA proteins are not identical in function but provide some degree of specificity to the COP1/SPA E3 ligase activity. The failure of PpSPAb and OsSPA1 to fully replace AtSPA1 in Arabidopsis supports that such functional divergence has occurred in the course of land plant evolution. While this is very reasonable, it is nevertheless significant that *COP1* coding sequences did not functionally co-diverge with *SPA* sequences, especially considering that both proteins carry very similar WD40-repeat domains in their C-termini which both are able to bind and thereby recognize the same substrate proteins [11]. Hence, COP1 must provide a core function to the COP1/SPA complex that hinders evolutionary divergence, and this core function is likely modified by divergent SPA proteins.

OsSPA1 was capable of restoring a wild-type phenotype in dark-grown *spa* triple mutant seedlings – though at low efficiency - but not in light-grown seedlings or adult plants. We consider two possible scenarios to explain this dark-specific complementation by OsSPA1: OsSPA1 activity may be reduced when compared to AtSPA1 and therefore be solely sufficient to restore SPA function in darkness but not in the light. This would be consistent with previous observations showing that mutations in a single *SPA* gene caused a mutant phenotype only in the light but not in darkness [24,55]. Hence, full SPA activity is much more critical in light-grown seedlings than in dark-grown seedlings, probably because the light-induced inactivation of the COP1/SPA complex causes additional stress on the activity of the complex. Alternatively, OsSPA1 may be hyperinactivated by Arabidopsis photoreceptors and, therefore, lack any activity in the light. This behavior is found in the Arabidopsis SPA2 protein which also shows high activity primarily in dark-grown seedlings [27]. Though the OsSPA1 sequence is more similar to Arabidopsis SPA1 than to SPA2, we do not exclude this possibility. Knocking out the *OsSPA1* gene in rice would allow to distinguish between these two possibilities.

The degree of complementation by *COP1* orthologs varied with respect to the different aspects of the *cop1* mutant phenotype. Most evidently, expression of OsCOP1 or PpCOP1a in light-grown *cop1-5* seedlings caused a reduced response to B and FR, a phenotype that is reminiscent of AtCOP1 *over*expression rather than of reduced COP1 activity. This observation suggests that OsCOP1 and PpCOP1a maintain higher activity in the light than AtCOP1 and are therefore incompletely inactivated by Arabidopsis photoreceptors when compared to the native Arabidopsis COP1 protein. Hence, photoreceptor and COP1 sequences appear to have co-evolved to allow optimal adaptation of seedling growth to the ambient light environment. When analyzing adult growth and development, PpCOP1a and OsCOP1 fully complemented the *cop1-5* leaf expansion phenotype while they only partially complemented the early-flowering phenotype of *cop1-5*. Since these phenotypes are mediated by distinct substrates, it is evident that the COP1-like proteins from rice and Physcomitrella do not polyubiquitinate all substrates of Arabidopsis COP1 equally well. Hence, functional conservation of COP1 may have varied with respect to the different substrates of COP1. Orthologs of known COP1/SPA substrates exist in *P. patens*, such as two PpHY5 and three PpCO-like (PpCOL) proteins [56-58]. A role of PpHY5 in moss light responses was described [58]. Hence, if PpCOP1a acts as a light-regulated ubiquitin ligase in mosses as well, it may indeed mediate degradation of the PpHY5 protein. In the future, it will be interesting to elucidate whether there is a COP1/SPA E3 ligase in Physcomitrella and, if so, which substrates are recognized.

## Conclusions

Our results show that COP1 protein sequences from Physcomitrella, rice and Arabidopsis are functionally conserved, while the sequences of the SPA proteins showed considerable functional divergence. This may - at least in part - reflect the fact that *COP1* is a single copy gene in flowering plants, while SPA proteins are encoded by a small gene family of two to four members, thus possibly allowing sub- or neofunctionalization. Light responses are very distinct in mosses and angiosperms [39]. Whether these differences reflect distinct signaling pathways including the recruitment of different transcription factors into the light signaling network needs to be resolved.

## Methods

### Sequences and ortholog prediction

Arabidopsis protein sequences correspond to the loci *SPA1* (At2g46340.1), *SPA2* (At4g11110.1), *SPA3* (At3g15354.1), *SPA4* (At1g53090.1) and *COP1* (At2g32950.1) of the annotated Arabidopsis Col genome TAIR10 annotation [59]. Rice proteins correspond to the loci OsCOP1 (Os02g53140.1), OsSPA1 (Os05g49590.1) and OsSPA3/4 (Os01g52640.3) of the Rice Genome Annotation Project Release 7 [60]. However, based on an amino acid sequence alignment with all other SPAs from Arabidopsis, Physcomitrella and rice, the corresponding reference sequence of OsSPA3/4 (Os01g52640.3) lacks a part of the WD40 repeat domain. Here we used an alternatively spliced sequence, Os01g524630.1, from Genome Annotation Project Release 5 which contains additional WD40 repeats and in our opinion reflects the full-length OsSPA3/4 protein. Physcomitrella proteins correspond to the loci indicated in Figure 1. They are derived from the *cosmoss.org* Physcomitrella patens V1.6 genome annotation [48].

To conduct a phylogentic reconstruction of the *COP1*/*SPA* genes in Arabidopsis, rice and Physcomitrella, first an all-against-all blast search was performed. To find homologous sequences between these species, blastp + version 2.2.9 [61] was used to build a blast database with protein sequences as indicated in Additional file 7: Table S1 and a blastp search was performed with an e-value cutoff of 10 by using the BLOSUM62 matrix. The resulting blastp results were then filtered by applying a changed version of formula (2) as indicated by [62]. These filtered blastp results were then used with proteinortho version 4.26 [63] to detect co-orthologs within and between these species by using the following options [-e = 0.01; -id = 11; -cov = 0.25; -conn = 0.1; -m = 0.75; -pairs; -selfblast; -blastdone]. The proteinortho results were filtered for COP1 (AT2G32950.1), SPA1 (AT2G46340.1), SPA2 (AT4G11110.1), SPA3 (AT3G15354.1), SPA4 (AT1G53090.1), RUP1 (AT5G52250.1), RUP2 (AT5G23730.1) and all resulting co-orthologs were used for further analysis and were screened for protein domains by InterProScan version 5 [64]. The program ncoils (based on [65]) is used by InterProScan with default settings to predict coiled-coils domains. In addition to standard settings we used different sliding window parameters [14,21,28] for the coiled-coils domain predictions which are highlighted in Additional file 3: Figure S3. For the phylogenetic reconstruction RUP1 and RUP2 were chosen as an outgroup gene family since both also contain WD40 repeats like the COP1/SPA genes but lack functional domains further upstream. These genes could be used to root COP1/SPA phylogenetic trees.

### Phylogenetic analysis

A multiple sequence alignment (MSA) was calculated with MAFFT L-INS-i version 7.037b [66], ProbCons version 1.12 [67], Muscle version 3.8.31 [68] and T-coffee version 8.99 [69] with default settings and subsequently combined into an optimal alignment using the combiner function of T-coffee. The MSA was visualized and manually curated using Jalview version 2.8 [70] (Additional file 2: Figure S2, Additional file 4: Figure S4, Additional file 5: Figure S5). The JTT + G + I + F model was selected as the best fitting amino acid substitution model according to the Bayesian Information Criterion in ProtTest version 3.3 [71]. To reconstruct the phylogeny we used MrBayes 3.2.2 [72] and RAxML version 8.0.2 [73].

For MrBayes we initiated two runs of four Markov-chain Monte Carlo (MCMC) chains of 2 × 10^7^ generations each from a random starting tree, sampling every 1,000 generations [additional settings: rates = invgamma, ngammacat = 4, aamodelpr = JTT]. A 25% burn-in was chosen and convergence was assessed by standard deviation of split frequencies falling below 0.005.

RAxML conducted 1,000 non-parametric bootstrap inferences with the rapid hill-climbing mode using the PROTGAMMAIJTTF model [additional settings: -d -b -#1000]. The bootstrap replicates were used to build a consensus tree applying the majority rule option (-m PROTGAMMAIJTTF -J MR). Phylogenetic trees were rooted by the RUP outgroup gene family and visualized with Figtree version 1.4.0 (http://tree.bio.ed.ac.uk/software/figtree/).

### Plant material, light sources and growth conditions

All mutant genotypes used were described previously: *cop1-4* (Col-0), *cop1-5* (Ws) [51], *spa1-7 spa3-1 spa4-1* and *spa1-7 spa2-1 spa3-1* (both Col) [34]. Light sources, seedling growth conditions and determination of seedling and adult traits were described previously [27].

### Plasmid constructions, plant transformations and selection of transgenic plants

All ORF clones were designed based on the sequence information provided in the databases described above. To generate *COP1* expression clones, *AtCOP1*, *OsCOP1* and *PpCOP1a* ORFs were amplified using gene-specific primers with attached attB sites and the amplified sequences were subsequently cloned into the pDONR221 entry vector by Gateway cloning according to the manufacturer´s instructions. *AtCOP1* and *PpCOP1a* ORFs were amplified from cDNA derived from Arabidopsis seedlings or Physcomitrella gametophores, respectively. *OsCOP1* was amplified from a full-length cDNA clone obtained from National Institute for Agrobiological Sciences (NIAS), Japan. The obtained Entry clones were recombined with the pGJ2169 GW binary destination vector (kindly provided by George Coupland) containing the *35S* promoter before the Gateway cassette. The final destination vectors were transformed into homozygous *cop1-4* and heterozygous *cop1-5/+* mutants. Transgenic plants were selected on Basta herbicide. In the *cop1-4* background, at least 25 independent transgenic lines per construct were analyzed in the T1 (flowering time, leaf size) and T2 (seedling deetiolation) generations. In the *cop1-5* background, lines homozygous for *cop1-5* and the respective transgene were generated by selecting for kanamycin resistance (*cop1-5*), the absence of the native *COP1* transcript and the presence of the introduced transgene (Basta resistance).

*SPA* expression clones were constructed as follows: First, 2260 bp or 1309 bp of the Arabidopsis *SPA1* or *SPA4* 5´ regulatory regions preceding the ATG start codon, respectively (*pSPA1*, *pSPA4*), were amplified from previously constructed plasmids using primers containing HindIII or SdaI restriction sites, respectively, and subsequently cloned into unique HindIII or SdaI restriction sites, respectively, of the pGWB1 destination vector [74]. These modified pGWB1 destination vectors now have Gateway cassettes after the *pSPA1* or *pSPA4* promoters, respectively. Second, Entry clones carrying the ORFs of *SPA* sequences were generated after amplifying the ORF of *AtSPA1* and *AtSPA4* from Arabidopsis cDNA, the *OsSPA1* ORF from a full-length cDNA clone obtained from NIAS, Japan, and the Physcomitrella *SPAb* ORF from cDNA synthesized from Physcomitrella gametophores (for all primer sequences, see Additional file 8: Table S2). Third, the modified pGWB1 destination vectors described above were recombined with the Entry clones containing the ORFs from *AtSPA1*, *OsSPA1*, *PpSPAb* and *AtSPA4*, respectively, using Gateway LR technology to generate *pSPA1::AtSPA1/OsSPA1/PpSPAb* vectors and *pAtSPA4::AtSPA4/PpSPAb* vectors. These binary vectors were transformed into Arabidopsis *spa1 spa3 spa4* and *spa1 spa2 spa3* mutants.

### RNA isolation and transcript analysis

RNA was isolated and reverse-transcribed as described previously [27]. *SPA* ORFs were amplified by semi-quantitative RT-PCR using gene-specific primers (Additional file 8: Table S2). PCR products were resolved by agarose electrophoresis and subsequent staining with ethidium bromide.

### Availability of supporting data

The data sets supporting the results of this article are included within the article and its additional files.

## Competing interests

The authors declare no competing interests.

## Authors’ contributions

AR, SD and UH carried out the molecular and genetic studies. KU and SR performed the sequence and phylogenetic analyses. All authors drafted, read and approved the final manuscript.

## Supplementary Material

Additional file 1: Figure S1 Phylogeny and domain structure of COP1 and SPA gene family in Arabidopsis, rice and Physcomitrella. **A**. Phylogenetic tree based on Bayesian inference created with COP1 and SPA homologs in three plant species. The Bayesian consensus phylogeny was constructed on a manual curated multiple sequence alignment rooted by the RUP gene family as an outgroup. Numbers on internal branches indicate Bayesian posterior probabilities. Line thickness corresponds to posterior probabilities. Detailed settings used for tree construction and tree plotting can be obtained from the methods chapter. **B**. Phylogenetic tree based on maximum likelihood created with COP1 and SPA homologs in three plant species. Consensus tree build by the majority rule of bootstrap replicates. Numbers on internal branches indicate support values of bootstrap in percent. Line corresponds to bootstrap support values. Detailed settings used for tree construction and tree plotting can be obtained from the methods chapter. **C**. Protein domains important for COP1 and SPA gene function obtained by InterProScan5. For each protein the domain structures obtained by InterProScan5 were plotted next to each protein. Individual domain position corresponds to their absolute position along the analyzed protein; red boxes, IPR013083 - Zinc finger, RING/FYVE/PHD - type; orange boxes, IPR001841 - Zinc finger, RING - type; light green boxes, IPR011009 - Protein kinase - like domain; green boxes, IPR000719 - Protein kinase domain; blue boxes, IPR015943/IPR017986 - WD40/YVTN repeat - like - containing domain; light blue boxes represent number of WD40 repeats, SM00320 - WD40 repeat; grey boxes represent number of coiled-coil occurrence based on Coils prediction. Click here for file

Additional file 2: Figure S2 Multiple sequence alignment of Arabidopsis, rice and Physcomitrella COP1 protein sequences. Sequence alignment displayed using Jalview version 2.8. Protein stretches belonging to InterProScan5 domain IPR001841 - Zinc finger, RING - type are highlighted in orange; predicted occurrence of coiled-coil domains are highlighted in grey; WD40 repeats, SM00320 - WD40 repeat are highlighted in light blue. Click here for file

Additional file 3: Figure S3 Prediction of coiled-coil domains in Arabidopsis, rice and Physcomitrella COP1 and SPA protein sequences. Prediction of coiled-coil domains were obtained from COILS (version 2.2) with three different sliding window parameters and the MTIDK matrix. Results indicating prediction probabilities for each window were plotted alongside the protein length. Next to each protein name obtained by the used sequence databases an alias was attached. Click here for file

Additional file 4: Figure S4 Multiple sequence alignment of Arabidopsis, rice and Physcomitrella SPA-related protein sequences. Sequence alignment displayed using Jalview version 2.8. Protein stretches belonging to InterProScan5 domain IPR011009 - Protein kinase - like domain are highlighted in light green; IPR000719 - Protein kinase domain are highlighted in green; predicted occurrence of coiled-coil domains are highlighted in grey; WD40 repeats, SM00320 - WD40 repeat are highlighted in light blue. Click here for file

Additional file 5: Figure S5 Multiple sequence alignment of Arabidopsis, rice and Physcomitrella RUP1-related protein sequences. Sequence alignment displayed using Jalview version 2.8. Protein stretches representing WD40 repeats, SM00320 - WD40 repeat are highlighted in light blue. Click here for file

Additional file 6: Figure S6 Hypocotyl elongation response of wild-type and transgenic *cop1-5* mutant seedlings to Rc (A) and FRc (B). Transgenic seedlings express *AtCOP1*, *OsCOP1* or *PpCOP1* under the control of the *35S* promoter. Two to three independent transgenic lines are shown. *cop1-5* mutant seeds failed to germinate due to the seedling-lethal phenotype and are therefore not shown. Error bars indicate the standard error of the mean (SEM). Click here for file

Additional file 7: Table S1 List of sequence databases used. Click here for file

Additional file 8: Table S2 Primer sequences. Click here for file
