# Supplementary figures and images for: Functional analysis of COP1 and SPA orthologs from Physcomitrella and rice during photomorphogenesis of transgenic Arabidopsis reveals distinct evolutionary conservation
Source: BMC Plant Biol. 2014 Jul 1;14:178. doi: 10.1186/1471-2229-14-178 (PMC4091655; doi:10.1186/1471-2229-14-178)

A

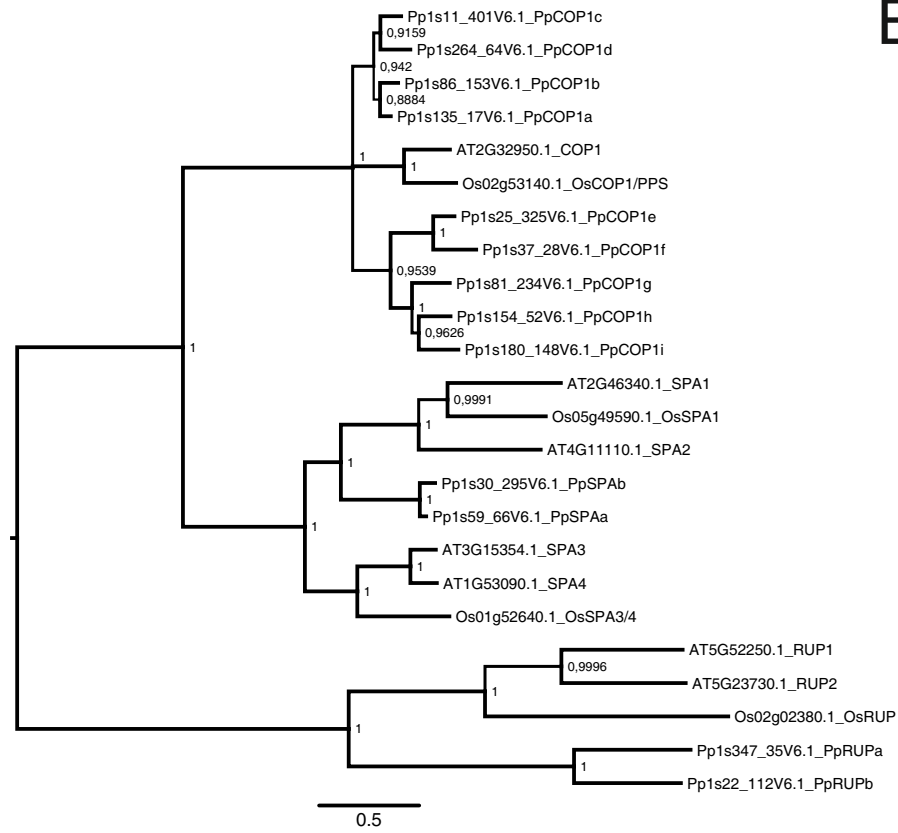

B

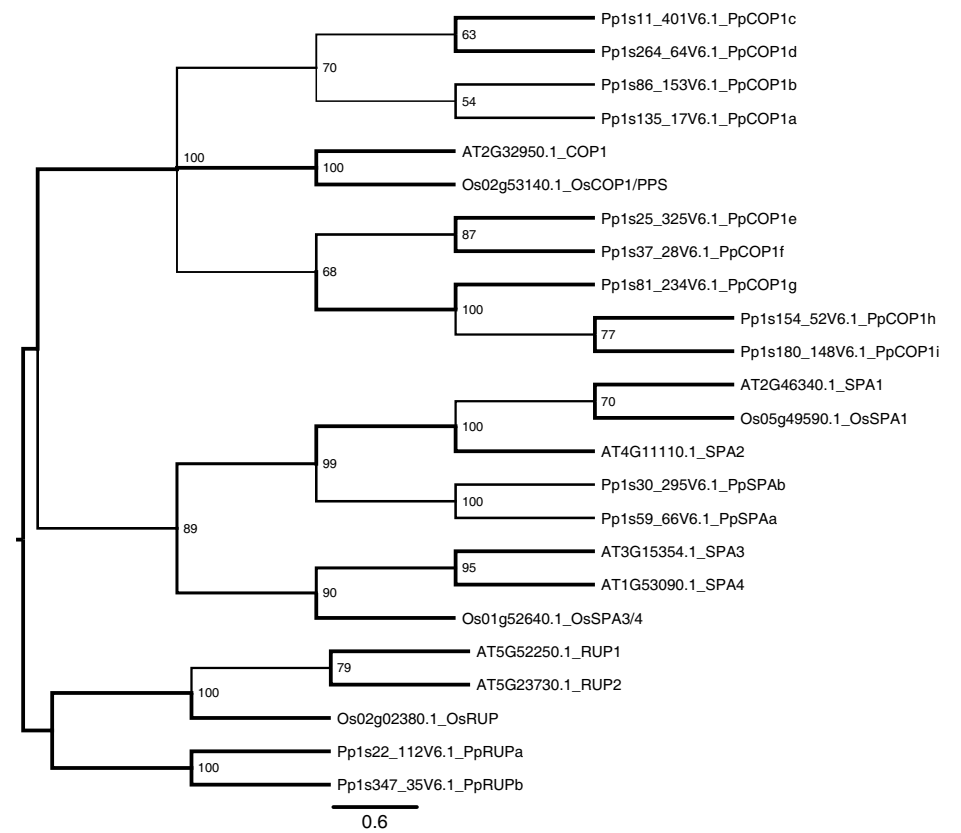

C

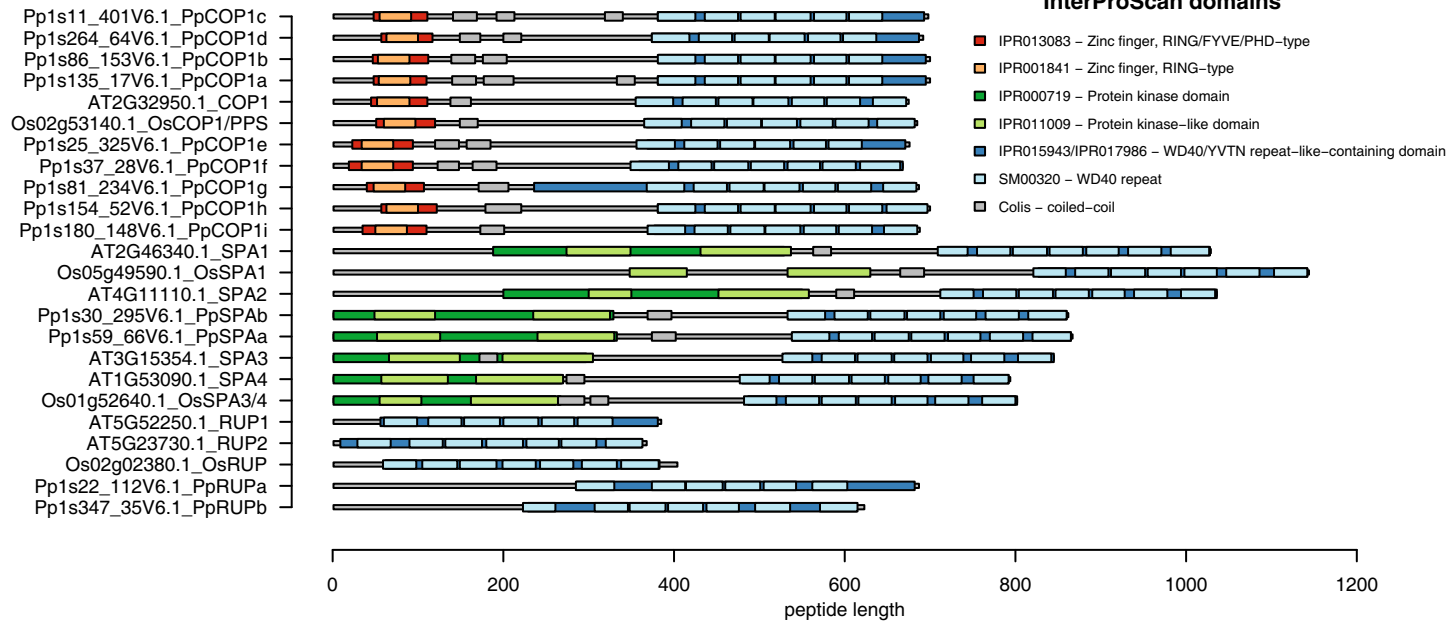

Supplement: Additional file 1: Figure S1 — Phylogeny and domain structure of COP1 and SPA gene family in Arabidopsis, rice and Physcomitrella. A. Phylogenetic tree based on Bayesian inference created with COP1 and SPA homologs in three plant species. The Bayesian consensus phylogeny was constructed on a manual curated multiple sequence alignment rooted by the RUP gene family as an outgroup. Numbers on internal branches indicate Bayesian posterior probabilities. Line thickness corresponds to posterior probabilities. Detailed settings used for tree construction and tree plotting can be obtained from the methods chapter. B. Phylogenetic tree based on maximum likelihood created with COP1 and SPA homologs in three plant species. Consensus tree build by the majority rule of bootstrap replicates. Numbers on internal branches indicate support values of bootstrap in percent. Line corresponds to bootstrap support values. Detailed settings used for tree construction and tree plotting can be obtained from the methods chapter. C. Protein domains important for COP1 and SPA gene function obtained by InterProScan5. For each protein the domain structures obtained by InterProScan5 were plotted next to each protein. Individual domain position corresponds to their absolute position along the analyzed protein; red boxes, IPR013083 - Zinc finger, RING/FYVE/PHD - type; orange boxes, IPR001841 - Zinc finger, RING - type; light green boxes, IPR011009 - Protein kinase - like domain; green boxes, IPR000719 - Protein kinase domain; blue boxes, IPR015943/IPR017986 - WD40/YVTN repeat - like - containing domain; light blue boxes represent number of WD40 repeats, SM00320 - WD40 repeat; grey boxes represent number of coiled-coil occurrence based on Coils prediction. [file 1471-2229-14-178-S1.pdf]

# COILS - prediction of coiled coil regions in COP1/SPA proteins (window size: 14; 21; 28 matrix: MTIDK)

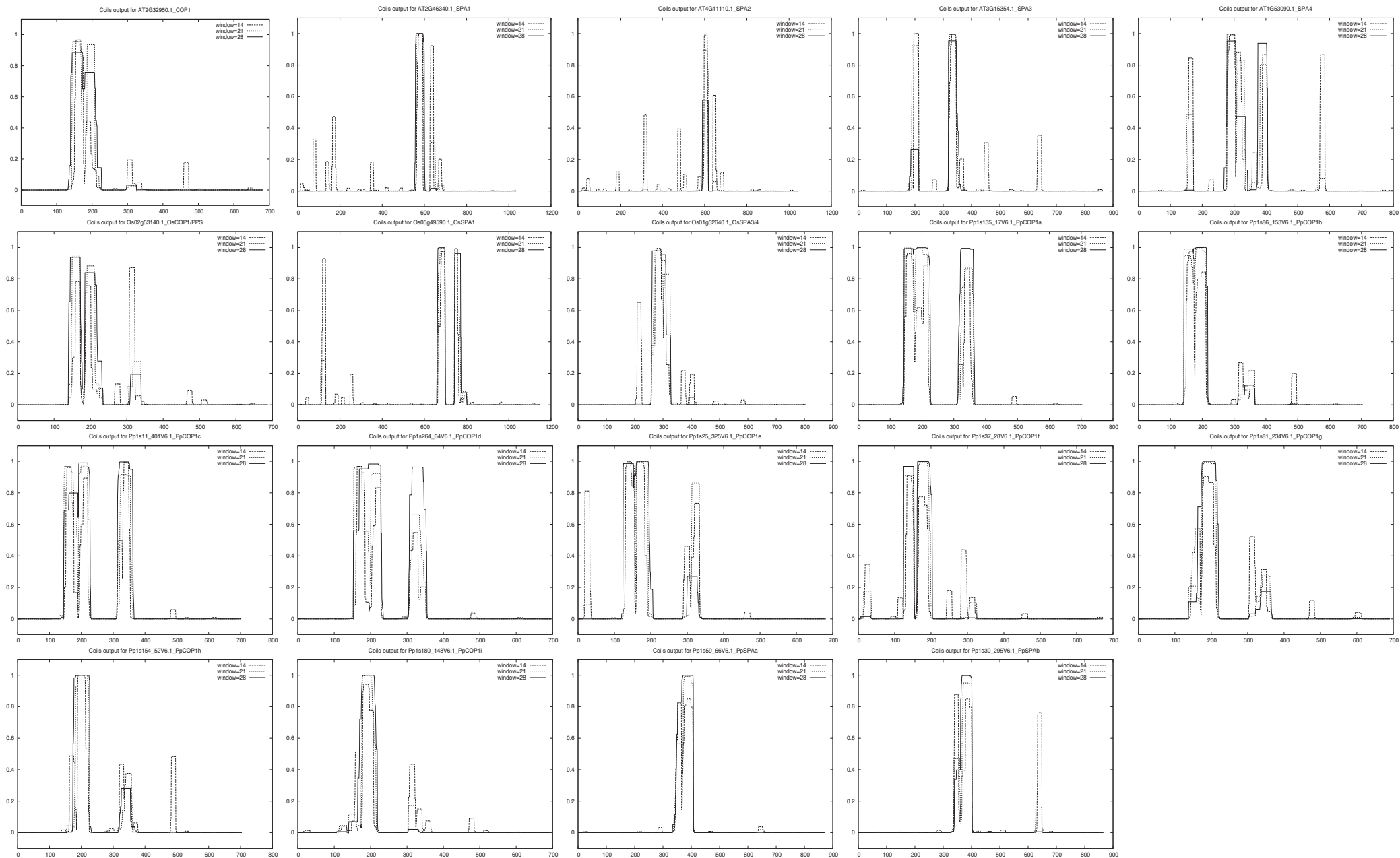

Supplement: Additional file 3: Figure S3 — Prediction of coiled-coil domains in Arabidopsis, rice and Physcomitrella COP1 and SPA protein sequences. Prediction of coiled-coil domains were obtained from COILS (version 2.2) with three different sliding window parameters and the MTIDK matrix. Results indicating prediction probabilities for each window were plotted alongside the protein length. Next to each protein name obtained by the used sequence databases an alias was attached. [file 1471-2229-14-178-S3.pdf]

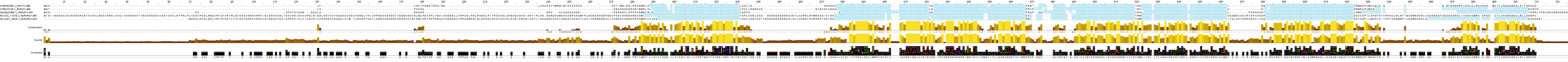

Supplement: Additional file 5: Figure S5 — Multiple sequence alignment of Arabidopsis, rice and Physcomitrella RUP1-related protein sequences. Sequence alignment displayed using Jalview version 2.8. Protein stretches representing WD40 repeats, SM00320 - WD40 repeat are highlighted in light blue. [file 1471-2229-14-178-S5.pdf]

**A**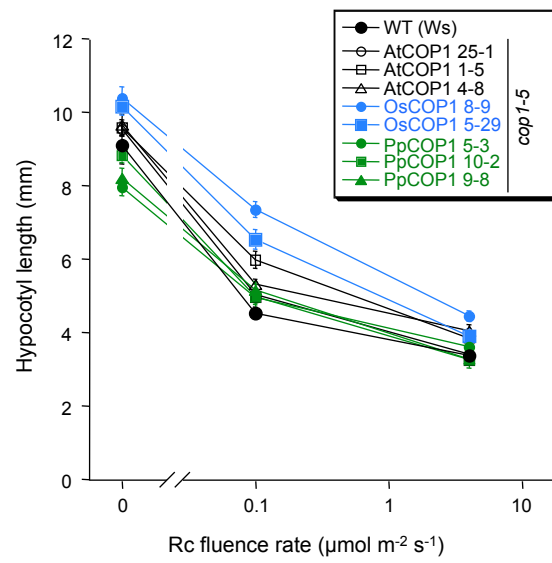**B**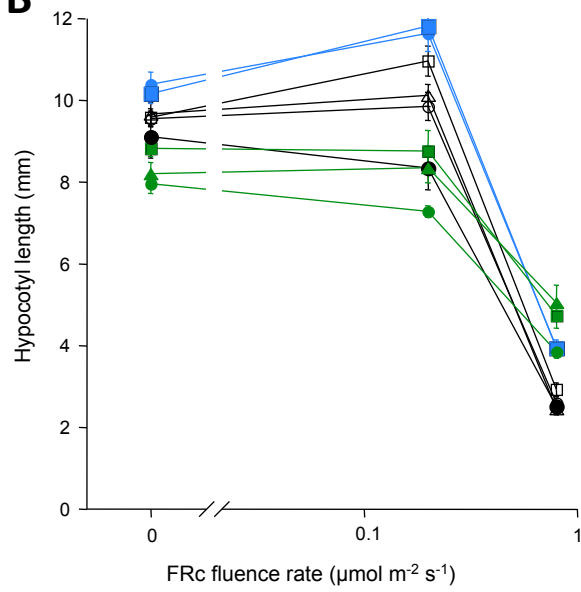

Supplement: Additional file 6: Figure S6 — Hypocotyl elongation response of wild-type and transgenic cop1-5 mutant seedlings to Rc (A) and FRc (B). Transgenic seedlings express AtCOP1, OsCOP1 or PpCOP1 under the control of the 35S promoter. Two to three independent transgenic lines are shown. cop1-5 mutant seeds failed to germinate due to the seedling-lethal phenotype and are therefore not shown. Error bars indicate the standard error of the mean (SEM). [file 1471-2229-14-178-S6.pdf]
