# Supplementary material for: Functional analysis of COP1 and SPA orthologs from Physcomitrella and rice during photomorphogenesis of transgenic Arabidopsis reveals distinct evolutionary conservation
Source: BMC Plant Biol. 2014 Jul 1;14:178. doi: 10.1186/1471-2229-14-178 (PMC4091655; doi:10.1186/1471-2229-14-178)
Supplement: Additional file 8: Table S2 — Primer sequences. [file 1471-2229-14-178-S8.docx]

Supplementary Table S2: Primer sequences

| **Oligonucleotides** | **Sequence (5´-> 3´)** | **Application** |
| --- | --- | --- |
| OsSPA1 GWFP | GGGGACAAGTTTGTACAAAAAAGCAGGCTT  CATGGCGGGGACGCATGGTTTTCG | *OsSPA1* ORF entry cloning |
| OsSPA1 GWRP | GGGGACCACTTTGTACAAGAAAGCTGGGT  ATCACACAAGCTCAAGCACTTTAATGC | *OsSPA1* ORF entry cloning |
| OsCOP1 GWFP | GGGGACAAGTTTGTACAAAAAAGCAGGCTT  CATGGGTGACTCGACGGTGGC | *OsCOP1* ORF entry cloning |
| OsCOP1 GWRP | GGGGACCACTTTGTACAAGAAAGCTGGGT  ATCAAGGAGCAAGTACAAGAACTT | *OsCOP1* ORF entry cloning |
| PpSPA17 attB1 | GGGGACAAGTTTGTACAAAAAAGCAGGCTA  TATGAAGGAGTTACCAGGCAG | *PpSPAb* ORF entry cloning |
| PpSPA17 attB2 | GGGGACCACTTTGTACAAGAAAGCTGGGT  ATCACACCATTTCCAAAATCTTG | *PpSPAb* ORF entry cloning |
| PpCOP1 attB1 | GGGGACAAGTTTGTACAAAAAAGCAGGCTA  TATGGAGGGAGGAGGTCCTTTC | *PpCOP1* ORF entry cloning |
| PpCOP1 attB2 | GGGGACCACTTTGTACAAGAAAGCTGGGT  ATCAGGGAGCAAGGACCAAGAC | *PpCOP1* ORF entry cloning |
| HinDIII-pAtSPA1F | AGACTAAGCTTAATAATACAACATGTTGCT  GGT | pAtSPA1-pGWB1 destination  vector generation |
| HinDIII-pAtSPA1R | CTAGAAAGCTTTAACAGGCATCAACACTCA  TT | pSPA1-pGWB1 destination  vector generation |
| SdaI-pSPA4F | AGACTCCTGCAGGATGATCTTCTTGGACAT  GCATC | pSPA4-pGWB1 destination  vector generation |
| SdaI-pSPA4R | CTAGACCTGCAGGTGATTACCAAACAAACT  CCTCT | pSPA4-pGWB1 destination  vector generation |
| AtSPA1-RT-F | GTGTTTTTCGAGGGGTTGTG | *AtSPA1* transgene expression |
| AtSPA1-RT-R | GACCAGGCTCTCTTCTGGTG | *AtSPA1* transgene expression |
| AtSPA4-RT-F | GGTCGGTCTTTAGCATTTGG | *AtSPA4* transgene expression |
| AtSPA4-RT-R | TGGAAATGCCTTGTGGTACA | *AtSPA4* transgene expression |
| OsSPA1-RT-F | GCTTCCTTTCTCAACTCAAGGA | *OsSPA1* transgene expression |
| OsSPA1-RT-R | ATTTTGACGAACCCTTAAAGCA | *OsSPA1* transgene expression |
| PpSPAb-RT-F | CCGCGAGTGACGAATTGAAAGC | *PpSPAb* transgene expression |
| PpSPAb-RT-R | CCCATCATCACTGCCGCTAGCTAAT | *PpSPAb* transgene expression |
| Act2-RT-F | ACTTTCATCAGCCGTTTTGA | *Actin* expression |
| Act2-RT-R | ACGATTGGTTGAATATCATCAG | *Actin* expression |
