# Supplementary material for: Functional analysis of COP1 and SPA orthologs from Physcomitrella and rice during photomorphogenesis of transgenic Arabidopsis reveals distinct evolutionary conservation
Source: BMC Plant Biol. 2014 Jul 1;14:178. doi: 10.1186/1471-2229-14-178 (PMC4091655; doi:10.1186/1471-2229-14-178)
Supplement: Additional file 2: Figure S2 — Multiple sequence alignment of Arabidopsis, rice and Physcomitrella COP1 protein sequences. Sequence alignment displayed using Jalview version 2.8. Protein stretches belonging to InterProScan5 domain IPR001841 - Zinc finger, RING - type are highlighted in orange; predicted occurrence of coiled-coil domains are highlighted in grey; WD40 repeats, SM00320 - WD40 repeat are highlighted in light blue. [file 1471-2229-14-178-S2.pdf]

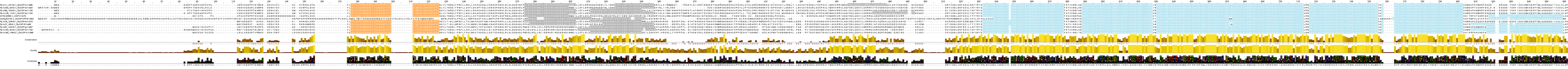
Conservation
Quality
Consensus
