# Supplementary material for: Functional analysis of COP1 and SPA orthologs from Physcomitrella and rice during photomorphogenesis of transgenic Arabidopsis reveals distinct evolutionary conservation
Source: BMC Plant Biol. 2014 Jul 1;14:178. doi: 10.1186/1471-2229-14-178 (PMC4091655; doi:10.1186/1471-2229-14-178)

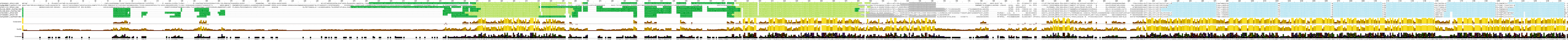

Supplement: Additional file 4: Figure S4 — Multiple sequence alignment of Arabidopsis, rice and Physcomitrella SPA-related protein sequences. Sequence alignment displayed using Jalview version 2.8. Protein stretches belonging to InterProScan5 domain IPR011009 - Protein kinase - like domain are highlighted in light green; IPR000719 - Protein kinase domain are highlighted in green; predicted occurrence of coiled-coil domains are highlighted in grey; WD40 repeats, SM00320 - WD40 repeat are highlighted in light blue. [file 1471-2229-14-178-S4.pdf]
